# Bis(4,4′-bipyridinium) di-μ-hydroxido-bis­[dihydroxido(pyridine-2,6-dicarboxyl­ato)anti­monate(III,V)] octa­hydrate

**DOI:** 10.1107/S1600536808001372

**Published:** 2008-01-23

**Authors:** Janet Soleimannejad, Hossein Aghabozorg, Yaghoub Mohammadzadeh Azar Golenji, Jafar Attar Gharamaleki, Harry Adams

**Affiliations:** a Department of Chemistry, Ilam University, Ilam, Iran; b Department of Chemistry, Teacher Training University, 49 Mofateh Avenue 15614, Tehran, Iran; c Department of Chemistry, Sheffield University, Sheffield S3 7HF, England

## Abstract

The reaction of anti­mony(III) chloride, 4,4′-bipyridine (4,4′-bipy) and pyridine-2,6-dicarboxylic acid (pydcH_2_), in a 1:2:2 molar ratio in an aqueous solution, resulted in the formation of the title centrosymmetric disordered mixed-valence Sb^III^/Sb^V^ compound, (C_10_H_9_N_2_)_2_[Sb_2_(C_7_H_3_NO_4_)_2_(OH)_6_]·8H_2_O or (4,4′-bipyH)_2_[Sb(pydc)(OH)_2_(μ-OH)]_2_·8H_2_O. The seven donor atoms of the (pydc)^2−^ groups and the hydroxido ligands form a distorted penta­gonal–bipyramidal arrangement around the Sb^III^/Sb^V^ centers. C—H⋯π stacking inter­actions between CH groups of the complex dianion and the aromatic rings of the (4,4′-bipyH)^+^ cations, with a distance of 2.89 Å, are observed. In the crystal structure, a wide range of noncovalent inter­actions, consisting of O—H⋯O, N—H⋯O and C—H⋯O hydrogen bonds [*D*⋯*A* ranging from 2.722 (2) to 3.137 (3) Å], ion pairing, π–π stacking [centroid–centroid distance of 3.4363 (13) Å] and C—H⋯π inter­actions, connect the various components into a supra­molecular structure.

## Related literature

For related literature, see: Aghabozorg, Attar Gharamaleki, Ghadermazi *et al.* (2007 ▶); Aghabozorg, Attar Gharamaleki, Ghasemikhah *et al.* (2007 ▶); Aghabozorg, Daneshvar *et al.* (2007 ▶).
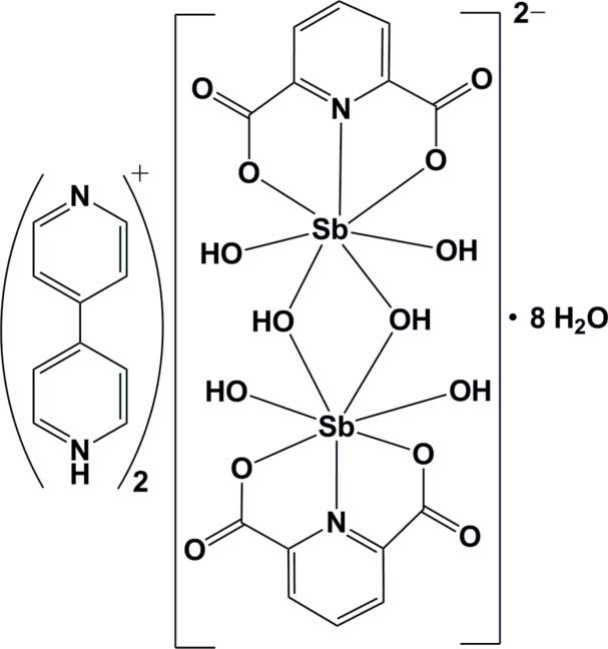


## Experimental

### 

#### Crystal data

(C_10_H_9_N_2_)_2_[Sb_2_(C_7_H_3_NO_4_)_2_(OH)_6_]·8H_2_O
*M* *_r_* = 1134.27
Triclinic, 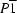

*a* = 10.0149 (11) Å
*b* = 10.4826 (12) Å
*c* = 11.0974 (12) Å
α = 92.816 (2)°
β = 97.813 (2)°
γ = 114.046 (2)°
*V* = 1047.0 (2) Å^3^
*Z* = 1
Mo *K*α radiation
μ = 1.38 mm^−1^
*T* = 150 (2) K
0.43 × 0.41 × 0.39 mm

#### Data collection

Bruker SMART 1000 diffractometer
Absorption correction: multi-scan (*SADABS*; Bruker, 2004 ▶) *T*_min_ = 0.588, *T*_max_ = 0.614 (expected range = 0.557–0.583)
12026 measured reflections
4790 independent reflections
4536 reflections with *I* > 2σ(*I*)
*R*_int_ = 0.022

#### Refinement

*R*[*F* ^2^ > 2σ(*F* ^2^)] = 0.023
*wR*(*F* ^2^) = 0.060
*S* = 1.07
4790 reflections
289 parameters
H-atom parameters constrained
Δρ_max_ = 0.91 e Å^−3^
Δρ_min_ = −0.70 e Å^−3^

### 

Data collection: *SMART* (Bruker, 2005 ▶); cell refinement: *SAINT* (Bruker, 2005 ▶); data reduction: *SAINT*; program(s) used to solve structure: *SHELXS97* (Sheldrick, 2008 ▶); program(s) used to refine structure: *SHELXL97* (Sheldrick, 2008 ▶); molecular graphics: *SHELXTL* (Sheldrick, 2008 ▶); software used to prepare material for publication: *SHELXTL*.

## Supplementary Material

Crystal structure: contains datablocks I, global. DOI: 10.1107/S1600536808001372/su2034sup1.cif

Structure factors: contains datablocks I. DOI: 10.1107/S1600536808001372/su2034Isup2.hkl

Additional supplementary materials:  crystallographic information; 3D view; checkCIF report

## Figures and Tables

**Table 1:** Hydrogen-bond geometry (Å, °)

*D*—H⋯*A*	*D*—H	H⋯*A*	*D*⋯*A*	*D*—H⋯*A*
O5—H5*A*⋯O10^i^	0.85	2.13	2.949 (2)	161
O6—H6*A*⋯O10^ii^	0.85	1.94	2.783 (2)	172
O7—H7*A*⋯O8^iii^	0.85	1.91	2.760 (2)	174
O8—H8*B*⋯O11^iv^	0.85	2.22	2.997 (2)	151
O8—H8*B*⋯O3^iv^	0.85	2.61	3.067 (2)	115
O8—H8*A*⋯N2	0.85	1.93	2.751 (2)	161
O9—H9*A*⋯O1^v^	0.85	1.91	2.749 (2)	170
O9—H9*B*⋯O6	0.85	1.88	2.731 (2)	177
O10—H10*A*⋯O11^vi^	0.85	2.03	2.867 (2)	168
O10—H10*B*⋯O9	0.85	1.87	2.722 (2)	178
O11—H11*A*⋯O4	0.85	1.95	2.793 (2)	174
O11—H11*B*⋯O8	0.85	1.99	2.830 (2)	169
N3—H3*A*⋯O6^vii^	0.85	1.91	2.760 (2)	173
C13—H13⋯O1^viii^	0.95	2.30	3.205 (3)	160
C15—H15⋯O5	0.95	2.23	3.107 (3)	153
C17—H17⋯O5^ix^	0.95	2.23	3.137 (3)	159
C5—H5⋯*Cg*1(N2/C11–C15)^x^	0.95	2.89	3.596 (2)	132

Symmetry codes: (i) 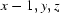; (ii) 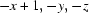; (iii) 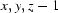; (iv) 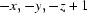; (v) 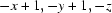; (vi) 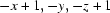; (vii) 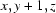; (viii) 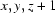; (ix) 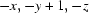; (x) 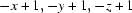.

**Fig. 1.**
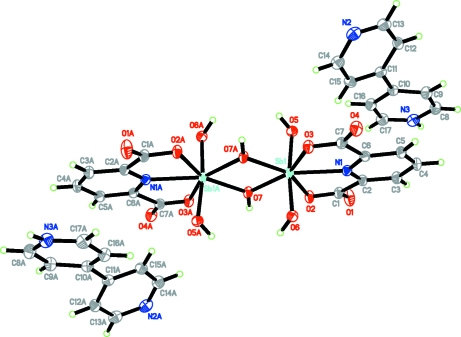
A view of the molecular structure of the title compound, showing the atom labelling scheme and displacement ellipsoids drawn at the 50% probability level. Uncoordinated water molecules are omitted for clarity. Atoms marked with A are related by the symmetry code (-x, -y, -z).

**Fig. 2.**
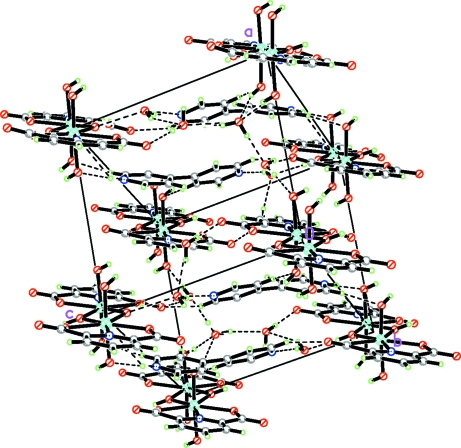
A view of the crystal packing of the title compound, with the hydrogen bonds shown as dashed lines.

**Fig. 3.**
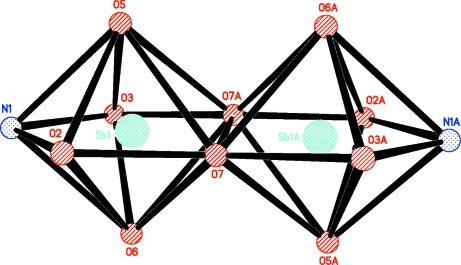
The coordination environment of the SbIII/SbV centers. Atoms marked with A are related by the symmetry code (-x, -y, -z).

**Fig. 4.**
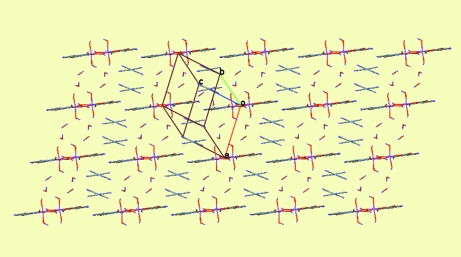
Layered diagram of the title compound. The space between the two layers of {[Sb(pydc)(OH)2(µ-OH)]2}2- dianions are filled with (4,4'-bipyH)+ cations and water molecules.

**Fig. 5.**
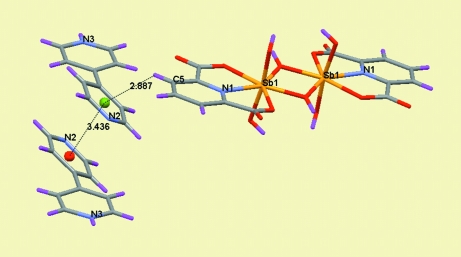
C—H···π stacking interactions between the C–H group of the (pydc)2– fragments and the aromatic rings of the (4,4'-bipyH)+ units. The C—H···π distance (measured to the center of phenyl ring) is 2.89 Å for C5—H5···Cg1 (1 - x, 1 - y, 1 - z). π-π stacking interactions between the aromatic rings of the (4,4'-bipyH)+ fragments with distance of 3.436 (2) Å for Cg1···Cg1 A (-x, 1 - y, 1 - z) [Cg1 and Cg1 A are the centroids of the rings N2/C11—C15 and N2A/C11A—C15A, respectively].

## supplementary crystallographic information

### Comment

Our research group has recently focused its attention on the one-pot synthesis of water soluble proton transfer compounds that can function as suitable ligands in the synthesis of metal complexes (Aghabozorg *et al.*, 2007*a*, Aghabozorg *et al.*, 2007*b*, Aghabozorg *et al.*, 2007*c*).

The title compound is compossed of a disordered mixed valent Sb^III^/Sb^V^ binuclear dianion, two protonated 4,4'-bipyridines, (4,4'-bipyH)^+^, and eight uncoordinated water molecules (Fig.1 and Fig. 2). The anionic Sb^III^/Sb^V^ binuclear complex is centrosymmetric; the binuclear units are related to one another by an inversion center, which lies at the center of the Sb_2_O_2_ four membered ring. Each antimony atom is coordinated to a tridentate (pydc)^2-^ ligand by the carboxylate O-atoms and the pyridine N-atom, and to two terminal hydroxo ligands. Two more hydroxo ligands also serve as bridges between the two Sb^III^/Sb^V^ centers. These bridging hydroxyl groups are remaining from SbOCl, formed during the partial hydrolysis of SbCl_3_. The Sb^III^/Sb^V ^centers have a distorted pentagonal bipyramidal environment (Fig. 3). Atoms O5 and O6 occupy the axial positions [O5—Sb1—O6 = 172.29 (6) °], whereas atoms N1, O2, O3, O7 and O7^i^ (i: -*x*, -*y*, -*z*) atoms form the equatorial plane.

In the crystal structure of the title complex, the spaces between layers of {[Sb(pydc)(OH)_2_(µ-OH)]_2_}^2-^ anions are filled with (4,4'-bipyH)^+^ cations and water molecules (Fig. 4). The dihedral angle between the two best-planes passing through the aromatic rings of (4,4'-bipyH)^+^ is 31.88 (17) °, which indicates the flexibility of the central C—C bond.

In the crystal structure of the title compound there are C—H···π stacking interactions between the C–H group of the (pydc)^2–^ fragments andthe aromatic rings of the (4,4'-bipyH)^+^ cations. The C—H···π distance (measured to the center of the pyridine ring) is 2.89 Å for C5—H5···*Cg*1 (1 - *x*, 1 - *y*, 1 - *z*) with an angle of 132 °. There are also π-π stacking interactions between the aromatic rings of the (4,4'-bipyH)^+^ cations, with a distance of 3.4363 (13) Å for *Cg*1···*Cg*1 A (-*x*, 1 - *y*, 1 - *z*) [*Cg*1 and *Cg*1 A are the centroids of rings N2/C11—C15 and N2A/C11A—C15A, respectively] (Fig. 5).

In the crystal structure, there are a wide range of non-covalent interactions, consisting O—H···O, N—H···O and C—H···O hydrogen bonds (Table 1 and Fig. 2), ion pairing, π–π and C—H···π stacking interactions (Table 1), all of which connect the various components into a supramolecular structure.

### Experimental

An aqueous solution (25 ml of water) of SbCl_3_ (290 mg, 1 mmol), 4,4'-bipyridine (310 mg, 2 mmol) and pyridine-2,6-dicarboxylic acid (360 mg, 2 mmol) was heated to boiling point for 2 h. Colorless crystals of the title compound were obtained from the solution after two days at room temperature.

### Refinement

The H-atoms were included in calculated positions and treated as riding atoms: O—H = 0.85 Å and C—H = 0.93 - 0.95 Å with *U*_iso_(H) = 1.2*U*_eq_(parent O or C-atom).

### Crystal data

**Table:**

(C_10_H_9_N_2_)_2_[Sb_2_(C_7_H_3_NO_4_)_2_(OH)_6_]·8H_2_O	*Z* = 1
*M**_r_* = 1134.27	*F*_000_ = 570
Triclinic, *P*1	*D*_x_ = 1.799 Mg m^−^^3^
Hall symbol: -P 1	Mo *K*α radiation λ = 0.71073 Å
*a* = 10.0149 (11) Å	Cell parameters from 9637 reflections
*b* = 10.4826 (12) Å	θ = 2.3–28.5º
*c* = 11.0974 (12) Å	µ = 1.38 mm^−^^1^
α = 92.816 (2)º	*T* = 150 (2) K
β = 97.813 (2)º	Block, colourless
γ = 114.046 (2)º	0.43 × 0.41 × 0.39 mm
*V* = 1047.0 (2) Å^3^	

### Data collection

**Table:**

Bruker SMART 1000 diffractometer	4790 independent reflections
Radiation source: fine-focus sealed tube	4536 reflections with *I* > 2σ(*I*)
Monochromator: graphite	*R*_int_ = 0.022
Detector resolution: 100 pixels mm^-1^	θ_max_ = 28.6º
*T* = 150(2) K	θ_min_ = 1.9º
ω scans	*h* = −13→13
Absorption correction: multi-scan(SADABS; Bruker, 2004)	*k* = −13→14
*T*_min_ = 0.588, *T*_max_ = 0.614	*l* = −14→14
12026 measured reflections	

### Refinement

**Table:**

Refinement on *F*^2^	Secondary atom site location: difference Fourier map
Least-squares matrix: full	Hydrogen site location: inferred from neighbouring sites
*R*[*F*^2^ > 2σ(*F*^2^)] = 0.023	H-atom parameters constrained
*wR*(*F*^2^) = 0.060	*w* = 1/[σ^2^(*F*_o_^2^) + (0.0357*P*)^2^ + 0.4561*P*] where *P* = (*F*_o_^2^ + 2*F*_c_^2^)/3
*S* = 1.07	(Δ/σ)_max_ = 0.001
4790 reflections	Δρ_max_ = 0.91 e Å^−^^3^
289 parameters	Δρ_min_ = −0.70 e Å^−^^3^
Primary atom site location: structure-invariant direct methods	Extinction correction: none

### Special details

**Table:**

Geometry. All e.s.d.'s (except the e.s.d. in the dihedral angle between two l.s. planes) are estimated using the full covariance matrix. The cell e.s.d.'s are taken into account individually in the estimation of e.s.d.'s in distances, angles and torsion angles; correlations between e.s.d.'s in cell parameters are only used when they are defined by crystal symmetry. An approximate (isotropic) treatment of cell e.s.d.'s is used for estimating e.s.d.'s involving l.s. planes.
Refinement. Refinement of *F*^2^ against ALL reflections. The weighted *R*-factor *wR* and goodness of fit *S* are based on *F*^2^, conventional *R*-factors *R* are based on *F*, with *F* set to zero for negative *F*^2^. The threshold expression of *F*^2^ > σ(*F*^2^) is used only for calculating *R*-factors(gt) *etc*. and is not relevant to the choice of reflections for refinement. *R*-factors based on *F*^2^ are statistically about twice as large as those based on *F*, and *R*- factors based on ALL data will be even larger.

### Fractional atomic coordinates and isotropic or equivalent isotropic displacement parameters (Å^2^)

**Table:**

	*x*	*y*	*z*	*U*_iso_*/*U*_eq_	
Sb1	0.140841 (13)	0.156744 (12)	0.066281 (11)	0.01594 (5)	
O1	0.26613 (19)	0.54127 (16)	−0.09185 (15)	0.0316 (4)	
O2	0.18376 (16)	0.31302 (14)	−0.06761 (13)	0.0199 (3)	
O3	0.23282 (16)	0.13870 (15)	0.26029 (13)	0.0199 (3)	
O4	0.40060 (16)	0.23914 (16)	0.43165 (13)	0.0250 (3)	
O5	0.00823 (16)	0.23727 (15)	0.12176 (13)	0.0195 (3)	
H5A	−0.0555	0.1808	0.1595	0.023*	
O6	0.29732 (15)	0.09324 (15)	0.02785 (13)	0.0191 (3)	
H6A	0.2826	0.0571	−0.0457	0.023*	
O7	−0.00038 (15)	0.04568 (14)	−0.09706 (12)	0.0176 (3)	
H7A	0.0135	0.0703	−0.1678	0.021*	
O8	0.02690 (17)	0.10749 (16)	0.66612 (14)	0.0263 (3)	
H8B	−0.0591	0.0577	0.6255	0.032*	
H8A	0.0522	0.1931	0.6543	0.032*	
O9	0.57770 (18)	0.19751 (17)	0.16030 (17)	0.0339 (4)	
H9A	0.6340	0.2752	0.1385	0.041*	
H9B	0.4909	0.1677	0.1186	0.041*	
O10	0.73724 (19)	0.04248 (19)	0.20370 (16)	0.0325 (4)	
H10A	0.7380	0.0232	0.2772	0.039*	
H10B	0.6871	0.0909	0.1924	0.039*	
O11	0.21742 (19)	0.02296 (18)	0.55043 (16)	0.0336 (4)	
H11A	0.2735	0.0927	0.5183	0.040*	
H11B	0.1591	0.0538	0.5768	0.040*	
N1	0.32909 (17)	0.36461 (17)	0.15357 (15)	0.0165 (3)	
N2	0.1174 (2)	0.3633 (2)	0.57504 (17)	0.0255 (4)	
N3	0.2738 (2)	0.87729 (19)	0.16815 (17)	0.0246 (4)	
H3A	0.2886	0.9474	0.1279	0.030*	
C1	0.2631 (2)	0.4435 (2)	−0.03335 (18)	0.0193 (4)	
C2	0.3588 (2)	0.4768 (2)	0.09189 (18)	0.0176 (4)	
C3	0.4662 (2)	0.6083 (2)	0.14286 (19)	0.0214 (4)	
H3	0.4875	0.6873	0.0982	0.026*	
C4	0.5418 (2)	0.6217 (2)	0.2608 (2)	0.0231 (4)	
H4	0.6172	0.7101	0.2973	0.028*	
C5	0.5066 (2)	0.5052 (2)	0.32512 (19)	0.0205 (4)	
H5	0.5555	0.5131	0.4065	0.025*	
C6	0.3983 (2)	0.3768 (2)	0.26789 (18)	0.0171 (4)	
C7	0.3418 (2)	0.2413 (2)	0.32655 (18)	0.0188 (4)	
C8	0.3600 (2)	0.9113 (2)	0.2791 (2)	0.0252 (4)	
H8	0.4367	1.0033	0.3014	0.030*	
C9	0.3369 (2)	0.8123 (2)	0.3604 (2)	0.0233 (4)	
H9	0.3985	0.8354	0.4384	0.028*	
C10	0.2220 (2)	0.6773 (2)	0.32763 (19)	0.0194 (4)	
C11	0.1893 (2)	0.5700 (2)	0.41423 (18)	0.0192 (4)	
C12	0.2114 (2)	0.6064 (2)	0.54032 (19)	0.0224 (4)	
H12	0.2522	0.7023	0.5737	0.027*	
C13	0.1729 (2)	0.5006 (2)	0.6161 (2)	0.0256 (4)	
H13	0.1864	0.5265	0.7016	0.031*	
C14	0.0983 (2)	0.3295 (2)	0.4541 (2)	0.0255 (4)	
H14	0.0601	0.2328	0.4238	0.031*	
C15	0.1307 (2)	0.4270 (2)	0.37092 (19)	0.0217 (4)	
H15	0.1136	0.3975	0.2857	0.026*	
C16	0.1364 (2)	0.6464 (2)	0.2104 (2)	0.0230 (4)	
H16	0.0589	0.5554	0.1853	0.028*	
C17	0.1649 (2)	0.7487 (2)	0.1313 (2)	0.0247 (4)	
H17	0.1079	0.7280	0.0514	0.030*	

### Atomic displacement parameters (Å^2^)

**Table:**

	*U*^11^	*U*^22^	*U*^33^	*U*^12^	*U*^13^	*U*^23^
Sb1	0.01651 (7)	0.01415 (7)	0.01362 (8)	0.00330 (5)	0.00071 (5)	0.00303 (5)
O1	0.0417 (9)	0.0180 (7)	0.0228 (8)	0.0030 (7)	−0.0058 (7)	0.0079 (6)
O2	0.0222 (7)	0.0156 (7)	0.0143 (7)	0.0012 (5)	−0.0006 (5)	0.0033 (5)
O3	0.0210 (7)	0.0179 (7)	0.0153 (7)	0.0035 (6)	−0.0007 (5)	0.0038 (5)
O4	0.0247 (7)	0.0272 (8)	0.0156 (7)	0.0050 (6)	−0.0027 (6)	0.0058 (6)
O5	0.0202 (7)	0.0199 (7)	0.0182 (7)	0.0080 (6)	0.0043 (5)	0.0032 (5)
O6	0.0186 (7)	0.0202 (7)	0.0172 (7)	0.0071 (6)	0.0022 (5)	0.0029 (5)
O7	0.0198 (6)	0.0148 (6)	0.0121 (6)	0.0012 (5)	0.0013 (5)	0.0049 (5)
O8	0.0286 (8)	0.0218 (7)	0.0206 (8)	0.0030 (6)	0.0021 (6)	0.0059 (6)
O9	0.0229 (8)	0.0237 (8)	0.0488 (11)	0.0052 (6)	−0.0033 (7)	0.0142 (7)
O10	0.0406 (9)	0.0425 (10)	0.0260 (8)	0.0269 (8)	0.0108 (7)	0.0095 (7)
O11	0.0363 (9)	0.0283 (9)	0.0356 (10)	0.0106 (7)	0.0111 (8)	0.0107 (7)
N1	0.0159 (7)	0.0166 (8)	0.0148 (8)	0.0047 (6)	0.0019 (6)	0.0021 (6)
N2	0.0231 (9)	0.0313 (10)	0.0240 (9)	0.0120 (8)	0.0047 (7)	0.0123 (8)
N3	0.0276 (9)	0.0237 (9)	0.0262 (10)	0.0129 (7)	0.0063 (7)	0.0108 (7)
C1	0.0199 (9)	0.0178 (9)	0.0163 (9)	0.0040 (7)	0.0018 (7)	0.0039 (7)
C2	0.0180 (9)	0.0169 (9)	0.0149 (9)	0.0047 (7)	0.0016 (7)	0.0021 (7)
C3	0.0221 (9)	0.0172 (9)	0.0211 (10)	0.0048 (8)	0.0021 (8)	0.0019 (8)
C4	0.0191 (9)	0.0184 (10)	0.0245 (11)	0.0023 (8)	0.0006 (8)	−0.0034 (8)
C5	0.0168 (9)	0.0242 (10)	0.0160 (9)	0.0054 (8)	−0.0008 (7)	−0.0010 (8)
C6	0.0151 (8)	0.0203 (9)	0.0150 (9)	0.0068 (7)	0.0018 (7)	0.0017 (7)
C7	0.0189 (9)	0.0193 (9)	0.0167 (9)	0.0062 (7)	0.0032 (7)	0.0028 (7)
C8	0.0248 (10)	0.0213 (10)	0.0281 (11)	0.0084 (8)	0.0033 (9)	0.0049 (9)
C9	0.0233 (10)	0.0229 (10)	0.0223 (10)	0.0093 (8)	0.0005 (8)	0.0037 (8)
C10	0.0201 (9)	0.0212 (10)	0.0185 (10)	0.0099 (8)	0.0036 (7)	0.0036 (8)
C11	0.0184 (9)	0.0233 (10)	0.0173 (9)	0.0099 (8)	0.0026 (7)	0.0046 (8)
C12	0.0204 (10)	0.0244 (10)	0.0184 (10)	0.0069 (8)	−0.0010 (8)	0.0003 (8)
C13	0.0224 (10)	0.0367 (12)	0.0170 (10)	0.0119 (9)	0.0016 (8)	0.0063 (9)
C14	0.0270 (10)	0.0224 (10)	0.0293 (12)	0.0115 (9)	0.0066 (9)	0.0072 (9)
C15	0.0247 (10)	0.0236 (10)	0.0180 (10)	0.0114 (8)	0.0037 (8)	0.0029 (8)
C16	0.0222 (10)	0.0240 (10)	0.0210 (10)	0.0084 (8)	0.0012 (8)	0.0043 (8)
C17	0.0252 (10)	0.0303 (11)	0.0195 (10)	0.0128 (9)	0.0013 (8)	0.0070 (9)

### Geometric parameters (Å, °)

**Table:**

Sb1—O5	1.9850 (14)	N3—C8	1.343 (3)
Sb1—O6	2.0211 (14)	N3—H3A	0.8500
Sb1—O7^i^	2.0898 (13)	C1—C2	1.513 (3)
Sb1—O7	2.0964 (13)	C2—C3	1.389 (3)
Sb1—O2	2.2169 (14)	C3—C4	1.390 (3)
Sb1—O3	2.2721 (14)	C3—H3	0.9500
Sb1—N1	2.2779 (16)	C4—C5	1.389 (3)
O1—C1	1.232 (3)	C4—H4	0.9500
O2—C1	1.276 (2)	C5—C6	1.390 (3)
O3—C7	1.279 (2)	C5—H5	0.9500
O4—C7	1.239 (2)	C6—C7	1.516 (3)
O5—H5A	0.8500	C8—C9	1.378 (3)
O6—H6A	0.8499	C8—H8	0.9500
O7—Sb1^i^	2.0898 (13)	C9—C10	1.403 (3)
O7—H7A	0.8499	C9—H9	0.9500
O8—H8B	0.8501	C10—C16	1.400 (3)
O8—H8A	0.8499	C10—C11	1.479 (3)
O9—H9A	0.8500	C11—C12	1.395 (3)
O9—H9B	0.8500	C11—C15	1.400 (3)
O10—H10A	0.8500	C12—C13	1.385 (3)
O10—H10B	0.8499	C12—H12	0.9500
O11—H11A	0.8501	C13—H13	0.9500
O11—H11B	0.8500	C14—C15	1.380 (3)
N1—C6	1.334 (3)	C14—H14	0.9500
N1—C2	1.337 (2)	C15—H15	0.9500
N2—C14	1.338 (3)	C16—C17	1.383 (3)
N2—C13	1.343 (3)	C16—H16	0.9500
N3—C17	1.342 (3)	C17—H17	0.9500
			
O5—Sb1—O6	172.26 (6)	C2—C3—C4	118.43 (19)
O5—Sb1—O7^i^	92.04 (6)	C2—C3—H3	120.8
O6—Sb1—O7^i^	92.06 (6)	C4—C3—H3	120.8
O5—Sb1—O7	96.38 (6)	C5—C4—C3	119.68 (19)
O6—Sb1—O7	91.19 (6)	C5—C4—H4	120.2
O7^i^—Sb1—O7	69.97 (6)	C3—C4—H4	120.2
O5—Sb1—O2	85.52 (6)	C4—C5—C6	118.62 (19)
O6—Sb1—O2	94.93 (6)	C4—C5—H5	120.7
O7^i^—Sb1—O2	144.33 (5)	C6—C5—H5	120.7
O7—Sb1—O2	74.95 (5)	N1—C6—C5	121.11 (18)
O5—Sb1—O3	93.16 (6)	N1—C6—C7	113.42 (17)
O6—Sb1—O3	81.44 (6)	C5—C6—C7	125.42 (18)
O7^i^—Sb1—O3	76.29 (5)	O4—C7—O3	126.20 (19)
O7—Sb1—O3	145.17 (5)	O4—C7—C6	119.09 (18)
O2—Sb1—O3	139.34 (5)	O3—C7—C6	114.70 (17)
O5—Sb1—N1	85.26 (6)	N3—C8—C9	119.8 (2)
O6—Sb1—N1	87.62 (6)	N3—C8—H8	120.1
O7^i^—Sb1—N1	145.09 (6)	C9—C8—H8	120.1
O7—Sb1—N1	144.94 (5)	C8—C9—C10	119.8 (2)
O2—Sb1—N1	70.26 (6)	C8—C9—H9	120.1
O3—Sb1—N1	69.14 (5)	C10—C9—H9	120.1
C1—O2—Sb1	120.96 (12)	C16—C10—C9	118.21 (19)
C7—O3—Sb1	122.17 (13)	C16—C10—C11	120.30 (18)
Sb1—O5—H5A	112.1	C9—C10—C11	121.48 (18)
Sb1—O6—H6A	114.1	C12—C11—C15	117.78 (19)
Sb1^i^—O7—Sb1	110.03 (6)	C12—C11—C10	121.82 (19)
Sb1^i^—O7—H7A	123.7	C15—C11—C10	120.38 (18)
Sb1—O7—H7A	124.2	C13—C12—C11	119.0 (2)
H8B—O8—H8A	109.6	C13—C12—H12	120.5
H9A—O9—H9B	110.3	C11—C12—H12	120.5
H10A—O10—H10B	107.5	N2—C13—C12	123.4 (2)
H11A—O11—H11B	102.1	N2—C13—H13	118.3
C6—N1—C2	120.88 (17)	C12—C13—H13	118.3
C6—N1—Sb1	120.49 (13)	N2—C14—C15	123.8 (2)
C2—N1—Sb1	118.34 (13)	N2—C14—H14	118.1
C14—N2—C13	117.14 (19)	C15—C14—H14	118.1
C17—N3—C8	122.71 (19)	C14—C15—C11	118.8 (2)
C17—N3—H3A	123.8	C14—C15—H15	120.6
C8—N3—H3A	113.1	C11—C15—H15	120.6
O1—C1—O2	125.97 (19)	C17—C16—C10	119.9 (2)
O1—C1—C2	118.90 (18)	C17—C16—H16	120.1
O2—C1—C2	115.12 (17)	C10—C16—H16	120.1
N1—C2—C3	121.23 (18)	N3—C17—C16	119.6 (2)
N1—C2—C1	113.09 (17)	N3—C17—H17	120.2
C3—C2—C1	125.67 (18)	C16—C17—H17	120.2
			
O5—Sb1—O2—C1	72.72 (15)	O1—C1—C2—C3	−8.7 (3)
O6—Sb1—O2—C1	−99.53 (16)	O2—C1—C2—C3	172.2 (2)
O7^i^—Sb1—O2—C1	159.97 (14)	N1—C2—C3—C4	−0.7 (3)
O7—Sb1—O2—C1	170.56 (16)	C1—C2—C3—C4	178.22 (19)
O3—Sb1—O2—C1	−16.93 (19)	C2—C3—C4—C5	−1.2 (3)
N1—Sb1—O2—C1	−13.85 (15)	C3—C4—C5—C6	1.6 (3)
O5—Sb1—O3—C7	−85.43 (15)	C2—N1—C6—C5	−2.0 (3)
O6—Sb1—O3—C7	88.99 (15)	Sb1—N1—C6—C5	−175.71 (14)
O7^i^—Sb1—O3—C7	−176.76 (16)	C2—N1—C6—C7	175.42 (17)
O7—Sb1—O3—C7	168.65 (14)	Sb1—N1—C6—C7	1.7 (2)
O2—Sb1—O3—C7	1.37 (19)	C4—C5—C6—N1	0.0 (3)
N1—Sb1—O3—C7	−1.72 (15)	C4—C5—C6—C7	−177.06 (19)
O5—Sb1—O7—Sb1^i^	−89.85 (7)	Sb1—O3—C7—O4	−178.17 (16)
O6—Sb1—O7—Sb1^i^	91.76 (7)	Sb1—O3—C7—C6	3.1 (2)
O7^i^—Sb1—O7—Sb1^i^	0.0	N1—C6—C7—O4	178.14 (18)
O2—Sb1—O7—Sb1^i^	−173.45 (8)	C5—C6—C7—O4	−4.6 (3)
O3—Sb1—O7—Sb1^i^	15.10 (13)	N1—C6—C7—O3	−3.1 (3)
N1—Sb1—O7—Sb1^i^	179.31 (7)	C5—C6—C7—O3	174.24 (19)
O5—Sb1—N1—C6	94.99 (15)	C17—N3—C8—C9	−0.7 (3)
O6—Sb1—N1—C6	−81.97 (15)	N3—C8—C9—C10	−1.0 (3)
O7^i^—Sb1—N1—C6	8.2 (2)	C8—C9—C10—C16	1.8 (3)
O7—Sb1—N1—C6	−170.64 (13)	C8—C9—C10—C11	−177.30 (19)
O2—Sb1—N1—C6	−178.07 (16)	C16—C10—C11—C12	−147.0 (2)
O3—Sb1—N1—C6	−0.22 (14)	C9—C10—C11—C12	32.1 (3)
O5—Sb1—N1—C2	−78.87 (15)	C16—C10—C11—C15	31.2 (3)
O6—Sb1—N1—C2	104.17 (15)	C9—C10—C11—C15	−149.8 (2)
O7^i^—Sb1—N1—C2	−165.64 (13)	C15—C11—C12—C13	−0.9 (3)
O7—Sb1—N1—C2	15.5 (2)	C10—C11—C12—C13	177.34 (19)
O2—Sb1—N1—C2	8.06 (14)	C14—N2—C13—C12	−0.6 (3)
O3—Sb1—N1—C2	−174.08 (16)	C11—C12—C13—N2	1.3 (3)
Sb1—O2—C1—O1	−161.95 (18)	C13—N2—C14—C15	−0.6 (3)
Sb1—O2—C1—C2	17.1 (2)	N2—C14—C15—C11	1.0 (3)
C6—N1—C2—C3	2.3 (3)	C12—C11—C15—C14	−0.2 (3)
Sb1—N1—C2—C3	176.18 (15)	C10—C11—C15—C14	−178.46 (19)
C6—N1—C2—C1	−176.70 (17)	C9—C10—C16—C17	−1.0 (3)
Sb1—N1—C2—C1	−2.9 (2)	C11—C10—C16—C17	178.12 (19)
O1—C1—C2—N1	170.32 (19)	C8—N3—C17—C16	1.5 (3)
O2—C1—C2—N1	−8.8 (3)	C10—C16—C17—N3	−0.6 (3)

Symmetry codes: (i) −*x*, −*y*, −*z*.

### Hydrogen-bond geometry (Å, °)

**Table:**

*D*—H···*A*	*D*—H	H···*A*	*D*···*A*	*D*—H···*A*
O5—H5A···O10^ii^	0.85	2.13	2.949 (2)	161
O6—H6A···O10^iii^	0.85	1.94	2.783 (2)	172
O7—H7A···O8^iv^	0.85	1.91	2.760 (2)	174
O8—H8B···O11^v^	0.85	2.22	2.997 (2)	151
O8—H8B···O3^v^	0.85	2.61	3.067 (2)	115
O8—H8A···N2	0.85	1.93	2.751 (2)	161
O9—H9A···O1^vi^	0.85	1.91	2.749 (2)	170
O9—H9B···O6	0.85	1.88	2.731 (2)	177
O10—H10A···O11^vii^	0.85	2.03	2.867 (2)	168
O10—H10B···O9	0.85	1.87	2.722 (2)	178
O11—H11A···O4	0.85	1.95	2.793 (2)	174
O11—H11B···O8	0.85	1.99	2.830 (2)	169
N3—H3A···O6^viii^	0.85	1.91	2.760 (2)	173
C13—H13···O1^ix^	0.95	2.30	3.205 (3)	160
C15—H15···O5	0.95	2.23	3.107 (3)	153
C17—H17···O5^x^	0.95	2.23	3.137 (3)	159
C5—H5···Cg1(N2/C11-C15)^xi^	0.95	2.89	3.596 (2)	132

Symmetry codes: (ii) *x*−1, *y*, *z*; (iii) −*x*+1, −*y*, −*z*; (iv) *x*, *y*, *z*−1; (v) −*x*, −*y*, −*z*+1; (vi) −*x*+1, −*y*+1, −*z*; (vii) −*x*+1, −*y*, −*z*+1; (viii) *x*, *y*+1, *z*; (ix) *x*, *y*, *z*+1;  (x) −*x*, −*y*+1, −*z*; (xi) −*x*+1, −*y*+1, −*z*+1.
